# Genetic Background Influence on Hippocampal Synaptic Plasticity: Frequency-Dependent Variations between an Inbred and an Outbred Mice Strain

**DOI:** 10.3390/ijms24054304

**Published:** 2023-02-21

**Authors:** Candice M. Roux, Pierre Lecouflet, Jean-Marie Billard, Elise Esneault, Marianne Leger, Pascale Schumann-Bard, Thomas Freret

**Affiliations:** 1 Department of Health, UNICAEN, INSERM, COMETE, CYCERON, Normandie University, 14000 Caen, France; 2 PORSOLT, 53940 Le Genest Saint-Isle, France

**Keywords:** synaptic plasticity, genetic background, hippocampus, electrophysiology, memory

## Abstract

For almost half a century, acute hippocampal slice preparations have been widely used to investigate anti-amnesic (or promnesic) properties of drug candidates on long-term potentiation (LTP)—a cellular substrate that supports some forms of learning and memory. The large variety of transgenic mice models now available makes the choice of the genetic background when designing experiments crucially important. Furthermore, different behavioral phenotypes were reported between inbred and outbred strains. Notably, some differences in memory performance were emphasized. Despite this, investigations, unfortunately, did not explore electrophysiological properties. In this study, two stimulation paradigms were used to compare LTP in the hippocampal CA1 area of both inbred (C57BL/6) and outbred (NMRI) mice. High-frequency stimulation (HFS) revealed no strain difference, whereas theta-burst stimulation (TBS) resulted in significantly reduced LTP magnitude in NMRI mice. Additionally, we demonstrated that this reduced LTP magnitude (exhibited by NMRI mice) was due to lower responsiveness to theta-frequency during conditioning stimuli. In this paper, we discuss the anatomo-functional correlates that may explain such hippocampal synaptic plasticity divergence, although straightforward evidence is still lacking. Overall, our results support the prime importance of considering the animal model related to the intended electrophysiological experiments and the scientific issues to be addressed.

## 1. Introduction

Hippocampal synaptic plasticity is widely known as a key cellular support for memory [1]. Almost half a century ago, long-term potentiation (LTP, i.e., an activity-dependent enhancement of synaptic transmission) was described in mammalian brains, first in vivo in the anesthetized rabbit [2], then shortly after ex vivo in guinea pig hippocampal slices [3]. Since these pioneer experiments, thousands of papers have been published on LTP, using both different experimental protocols and biological materials [4].

Among existing conditioning protocols, high-frequency stimulation (HFS) and theta-burst stimulation (TBS) are the two electrical stimulations that have been the most widely used to induce LTP within the CA1 area of the hippocampus [1,5]. Both mimic naturally occurring hippocampal electric oscillations. Respectively described as gamma -γ- (30–100 Hz) and theta -θ- (4–12 Hz) rhythms, these oscillatory frequencies are observed during spatial and contextual learning [6,7,8]. Therefore, considering the pivotal role of hippocampal LTP in memory, it became common to correlate changes in LTP strength with behavioral performance in hippocampal-dependent tasks [9]. The HFS protocol—as the first historically described pattern of LTP induction—still remains the most often used, but the TBS pattern appears to be favored when investigating modulations of neurotransmission systems, particularly the GABAergic tone (GABA: γ-aminobutyric acid) [5].

Although the first LTP experiment was performed on a rabbit, most works carried out since in neuroscience research have been conducted in mice [10]. Their use has enabled major breakthroughs over the last decades, notably in the understanding of memory processes and for better management of neurological diseases with memory impairments [10]. To this end, hundreds of different mice strains, resulting from careful breeding for the selection of desirable phenotypes, have been designed. This large variety of mice strains now offers a wealth of choices of biological materials, but raises, at the same time, the question of biological variability between genotypes. The same holds true when considering the nature of neurobiological mechanisms in LTP induction and development.

Historically, pharmacological and behavioral research used outbred mice strains [11,12], defined as a closed population (for at least four generations) of genetically variable mice, thus displaying a high level of heterozygosity [13]. However, following the intensive and rapid development of genetic engineering, inbred mice (stemming from 20 consecutive generations of sibling mating) became by far the most popular choice for scientists [14]. Inbred strains yield a stable genetic background for the generation of a wealth of transgenic mice. As early as the 1920s, several lines of evidence started to demonstrate the profound influence of the genetic background on behavioral outcomes. Previously, synaptic plasticity correlates of memory were mainly investigated between inbred strains or sub-strains [9,15] and only one study aimed at comparing inbred and outbred strains of mice [16]. Hence, the research community ran a number of studies on both strain subtypes with little awareness of the importance of strain selection while interpreting the results. Among the most widely used inbred and outbred mice subtypes are C57BL/6 and NMRI mice, respectively. Few studies were aimed at comparing the memory capacities of these two strains, and the expression of synaptic plasticity had not yet been investigated. Hence, the objective of this study was to investigate and compare the functional synaptic plasticity at CA3/CA1 hippocampal synapses of inbred C57BL/6 strains and outbred NMRI stock.

## 2. Results

**Figure 1:**
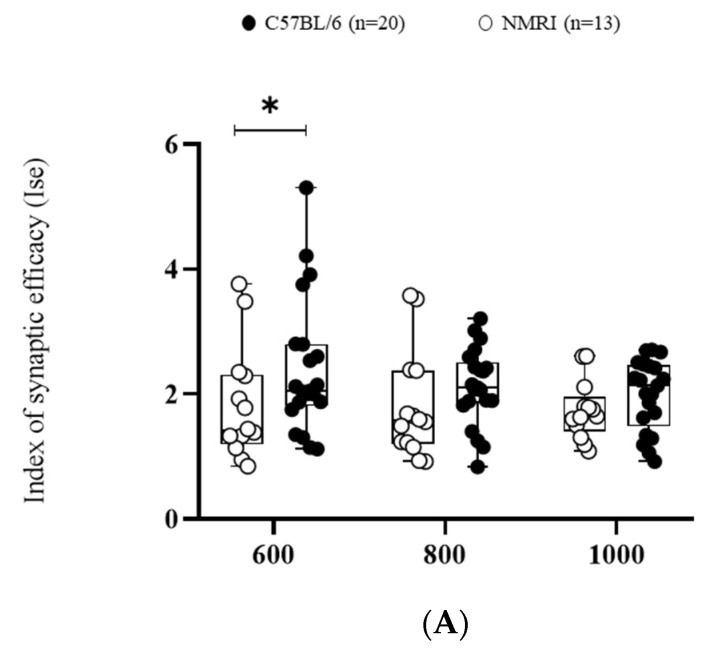

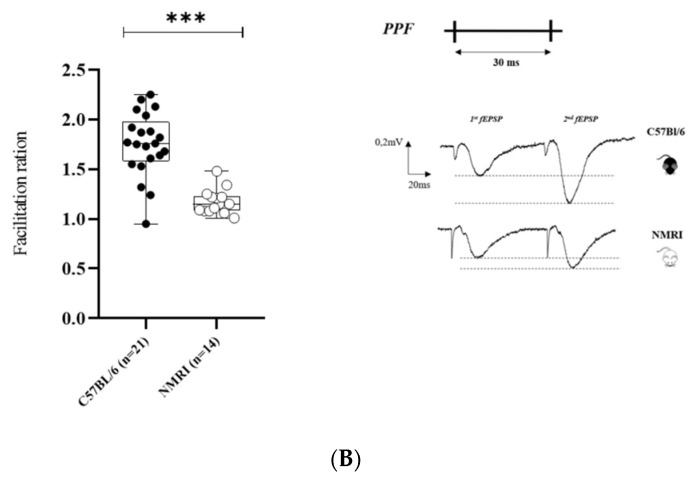
Basal synaptic transmission and paired-pulse facilitation are higher in C57BL/6 than in NMRI. (**A**) Basal synaptic transmission determined by I/O curves in slices from C57BL/6 mice (*n* = 20 slices/*n* = 14 mice) and NMRI mice (*n* = 13). Data are expressed as median ± interquartile. ANOVA for repeated measures (* *p* < 0.05). (**B**) Facilitation ratio in slices from C57BL/6 mice (*n* = 21 slices/*n* = 14 mice) and NMRI mice (*n* = 14 slices/*n* = 11 mice) (left). Stimulation pattern of PPF with corresponding representative traces of paired-pulse-induced fEPSP from NMRI and C57BL/6 mice (right). Each 2nd fEPSP slope was divided by the slope of the 1st fEPSP. Data are expressed as median ± interquartile. Mann–Whitney U test (*** *p* < 0.001).

First of all, the efficacy of basal synaptic transmission was measured using the index of synaptic efficiency (ISE) corresponding to the field excitatory post-synaptic potential (fEPSP) slope/pre-synaptic fiber volley (Fv). This ratio significantly differed between strains (F_(1,∞)_ = 5.3249, * *p* < 0.05). C57BL/6 exhibited a higher ISE compared to NMRI mice at the lowest stimulation intensity (600 mV) (Figure 1A).

Pre-synaptic short-term plasticity reflected by paired-pulse facilitation (PPF) was also found to be decreased in NMRI compared to C57BL/6, as shown by the decreased facilitation index (*** *p* <0.001) (Figure 1B).

**Figure 2:**
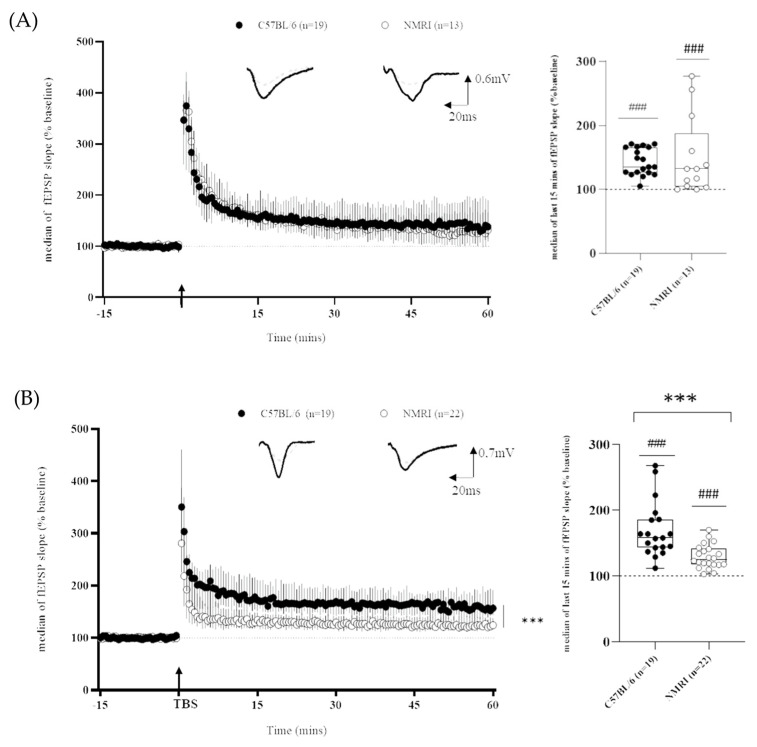
Differential LTP expression in outbred and inbred mice strains depends on stimulating frequency. (**A**) Time course of fEPSP slope after HFS-induced LTP in slices from C57BL/6 mice (*n* = 19 slices/*n* = 14 mice) and NMRI mice (*n* = 13 slices/*n* = 10 mice) (**left**) and corresponding last 15 min of fEPSP slope (**right**). (**B**) Time course of fEPSP slope after TBS-induced LTP in slices from C57BL/6 mice (*n* = 19 slices/*n* = 13 mice) and NMRI mice (*n* = 22 slices/*n* = 18 mice) (**left**) and corresponding last 15 min of fEPSP slope (**right**). Data are expressed as median ± interquartile. Arrow marks the time when conditioning stimulation was applied. Insets show representative traces of fEPSP before (dashed line) and after (full line) conditioning stimulation (univariate test, ### *p* <0.001: last 15 min of recording versus theoretical value of 100%; ANOVA for repeated measures *** *p* <0.001 and Mann–Whitney U test *** *p* < 0.001 for inbred versus outbred comparison).

As regards long-term plasticity recordings, it is worth mentioning that, independently of the mice strain, LTP was successfully induced within the CA1 field, as shown by the significant difference between the baseline and the last 15 min of recordings (Figure 2) using both the HFS protocol (C57BL/6: 143 ± 4%; ### *p* < 0.001, *n* = 19 and NMRI: 149 ± 18%, *n* = 13; ### *p* < 0.001 versus the theoretical value of 100%) and the TBS protocol (C57BL/6: 169 ± 10%, ### *p* < 0.001, *n* = 19 and NMRI: 127 ± 4%, *n* = 22; ### *p* < 0.001, vs. the theoretical value of 100%).

When making an inter-strain comparison, using the HFS protocol (Figure 2A), the LTP magnitude did not differ between strains (143 ± 4% versus 149 ± 18% for C57BL/6 and NMRI, respectively). Nevertheless, the coefficient of variation (CV), which is an index of within-strain variability, was higher in NMRI mice than in C57BL/6 (*** *p* < 0.0001, cf. Table 1).

Conversely, the magnitude of TBS-induced LTP was higher in hippocampal slices of C57BL/6 mice than in NMRI ones (169 ± 10% versus 127 ± 4%). Statistical analysis of the last 15 min of fEPSP recordings revealed a strain effect (F_(1,∞)_ = 24.7450, *** *p* < 0.001). No significant effect of time (F_(11,∞)_ = 1.4467, *p* = 0.1415) or group × time interaction (F_(11,∞)_ = 0.9134, *p* = 0.5283) was detected (Figure 2B). The strain difference was confirmed when the medians of the last 15 min of recording were compared between groups (Mann-Whitney, *** *p* < 0.001). Furthermore, NMRI displayed a lower CV than C57BL/6 mice (*** *p* < 0.001, cf. Table 1).

**Figure 3:**
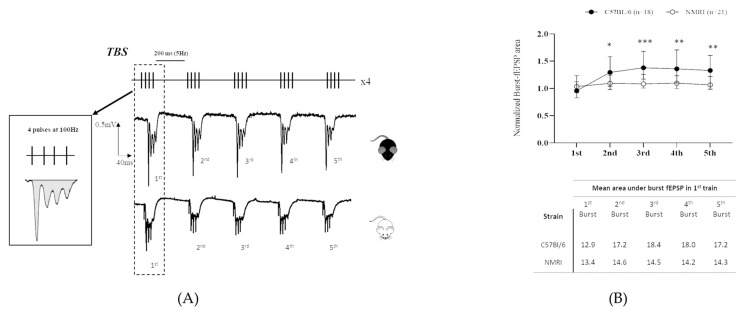
C57BL/6 mice exhibit higher degree of facilitation than NMRI outbred stock during TBS. (**A**) Typical TBS pattern of stimulation with corresponding sample trace of burst-induced fEPSP from NMRI and C57BL/6 mice. Area of burst-induced fEPSPs was measured as the total fEPSP area marked in shadow. (**B**) Normalized burst-fEPSP areas from C57BL/6 mice (*n* = 18 slices/*n* = 12 mice) and NMRI mice (*n* = 21 slices/*n* = 17 mice). Each burst-fEPSP was normalized to the first one. Data are expressed as median ± interquartile. Mann–Whitney U test for comparison of 2nd vs. 1st burst and for comparison between C57BL/6 and NMRI groups: * *p* < 0.05, ** *p* < 0.01, *** *p* < 0.001.

Interestingly, the facilitation (AUC of second burst relative to the first one) was clearly marked in C57BL/6 mice (### *p* < 0.001), while it was more discreet and non-significant in NMRI mice (Figure 3A).

Thus, consistent with their higher exhibited TBS-LTP magnitude, C57BL/6 mice also showed a higher degree of facilitation across bursts compared to NMRI mice (* *p* < 0.05; *** *p* < 0.001) (Figure 3B).

## 3. Discussion

Hippocampal synaptic excitability/plasticity is often discussed independently of mice-strain considerations. Here, we showed for the first time the existence of different electrophysiological profiles from the dorsal hippocampus between inbred and outbred mice strains and therefore shed light on the need to pay attention to the genetic background.

Basal hippocampal synaptic transmission was found to be higher in C57BL/6 mice than in NMRI mice. This electrophysiological parameter reflects glutamate transmission (i.e., neurotransmitter release and subsequent AMPA receptor activity/recruitment and/or densities). Similarly, functional short-term plasticity (PPF) was higher in C57BL6 mice compared to outbred NMRI mice. PPF reflects presynaptic calcium signaling that influences the probability of neurotransmitter release (Pr). During the second stimulus, Pr depends directly on the remaining (after the first stimulus) presynaptic stock of free calcium. Therefore, the higher the stock, the higher the Pr will be, but the lower the PPF will be. To sum up, these first results demonstrated that C57BL/6 mice are more prone (than NMRI ones) to presynaptic discharge in response to electrical stimuli.

Finally, we unveiled long-term plasticity differences using two conditioning protocols (TBS and HFS). While both mice strains displayed comparable levels of LTP following the HFS protocol, C57Bl6 mice displayed a higher level of TBS-induced LTP compared to NMRI mice.

At the post-synaptic site, the *N*-methyl-_D_-aspartate receptor subtype of glutamate receptors (NMDA-R) is critical to LTP induction, and previous experiments interestingly showed genetic differences in NMDA-R/glutamate receptor–channel complex expression [17]. However, this is unlikely to explain our results, as no difference was noted when using the HFS-induced LTP protocol (which also relies on NMDA-R). A deeper investigation of plasticity-related proteins or molecules that are preferentially involved in TBS would be of interest. An LTP deficit in inbred DBA/2 mice was, for instance, previously shown to correlate with a decrease in hippocampal protein kinase C compared with the C57BL/6 mice strain (Matsuyama et al., 1997). Similarly, given that pyramidal cell excitability is a determinant for LTP induction, it would be worth investigating underlying molecular mechanisms, such as type 5 metabotropic glutamate receptors (mGluR5) [18], potassium channels [19] or frequency-dependent cell responsiveness [20].

Of note, our results are somewhat consistent with previous data from the literature. For instance, LTP differences between C57BL/6 and other inbred sub-strains studied were strikingly pronounced when using the TBS protocol, whereas they were more subtle when following the HFS protocol [9].

Despite the number of studies performed in one or another strain subtype, differences between inbred and outbred strains have been, and unfortunately remain, poorly investigated. Few anatomical differences were identified in inbred C57BL/6 when compared with outbred NMRI, the most striking differences being a higher density of both the mossy fiber layer (MF) and excitatory dentate granule cells, as well as longer infra-pyramidal MF projections [21,22,23]. In a way, these anatomical differences are in accordance with the highest TBS-induced LTP magnitude observed in C57BL6 mice. According to the hippocampus tri-synaptic loop model, the CA1 neuronal response, as we measured it, should be shaped by both the dentate gyrus (DG) and MF, which, respectively, act upstream as either a preprocessor or detonator [24,25].

Conversely, these anatomical differences are at odds with a similar level of HFS-induced LTP. However, this similarity could easily be explained by the common hippocampal neurochemical profile and histochemical pattern between strains of mice [26]. Nevertheless, this argument should be taken with caution since not all neurotransmission systems have been investigated so far. For instance, the GABAergic system is clearly differently involved in the two LTP conditioning protocols [27]. Contrary to HFS, TBS-induced LTP closely relies on pyramidal cell disinhibition, which is dependent on GABAergic neurotransmission. The feedforward inhibition of pyramidal cells (“priming”) that occurs early during induction of LTP is then suppressed through the auto-inhibition process for about a second. With respect to 200 ms inter-burst intervals, these ensure maximal postsynaptic pyramidal cell depolarization, hence resulting in the highest LTP magnitude. Therefore, the efficacy of TBS-induced LTP (LTP magnitude) relies on the degree of facilitation that can be estimated through the measurement of the area under the curve (AUC) during the five bursts in the first train of stimulation. Interestingly, we showed that the higher exhibited TBS-LTP magnitude in C57BL/6 mice was consistent with a higher degree of facilitation across bursts compared to NMRI. Unfortunately, a clear-cut conclusion is dampened by the few comparative studies regarding the genetic background’s influence on neurotransmission systems, including the GABAergic one. Since the outcome of GABAergic inhibition could be interpreted multi-fold depending on the receptors targeted (either all chloride channels by picrotoxin, post-synaptic GABA_A_-Rs or pre- or post-synaptic GABA_B_-Rs), further comparative studies may benefit from the investigation of GABA-R densities and their morphology as well as their functional properties (i.e., conductance, membrane resistance, capacitance, etc.).

Ultimately, our work also raises the question of whether or not LTP induced artificially could be considered a model for learning mechanisms. In other words, whether or not the slight discrepancies in ex vivo hippocampal synaptic plasticity between strains affect behavioral performance, especially when spatial cognitive functions are involved. Unfortunately, comparisons between behavioral studies are scarce, and results remain partly divergent. To the best of our knowledge, four studies have so far compared spatial memory performances (Morris water maze) in C57BL/6 versus NMRI mice (see Table 1). Most often, a similar level of performance (both during the learning phase and probe test) is described [28,29,30,31], thus in agreement with ex vivo results for HFS-LTP. In previous research, when notable, the discrepancy was in favor of NMRI mice, which displayed a faster learning rate [30,31]. This last result contrasts with the higher level of TBS-LTP observed in C57BL/6 mice. The MWM test already proved to be sensitive to D-AP5, an NMDA-R antagonist (D,L-2-amino-5-phosphonovaleric acid) also known to induce an LTP deficit [32,33]. Consequently, this test is appropriate to compare memory performance between two strains in line with their electrophysiological pattern. However, the aversive character of water should be kept in mind, as it could interfere with the result as soon as a strain-different sensitivity to stress can be highlighted [34]. In addition, dissociation between memory performances and the LTP level has been described several times [35,36]. Although useful to catch underlying molecular mechanisms of the memory process, a direct correlation of LTP level (either ex vivo or in vivo) with learning performance (as just done) is not always true and is often a too simplistic way of thought. Neither are there only two types of LTP, nor only one brain structure involved in learning and memory processes.

## 4. Materials and Methods

### 4.1. Animals

All experiments were carried out in accordance with the European Community guidelines (2010/63EU) and the French law on animal experimentation. Electrophysiological experiments were conducted on dorsal hippocampal slices obtained from adult male mice aged 3–6 months from either C57BL6/Rj or NMRI strains (respectively, 28 ± 0.8 g and 29 ± 0.3 g) (purchased from Janvier Labs, France). Mice were housed in groups of 8 in standard polycarbonate cages, with food and water given ad libitum. The animal facility was under a reversed 12:12 light–dark cycle (light off at 7am), with a controlled environment in temperature (22 ± 1 °C) and hygrometry (55 ± 10%).

### 4.2. Electrophysiological Recordings

**Figure 4:**
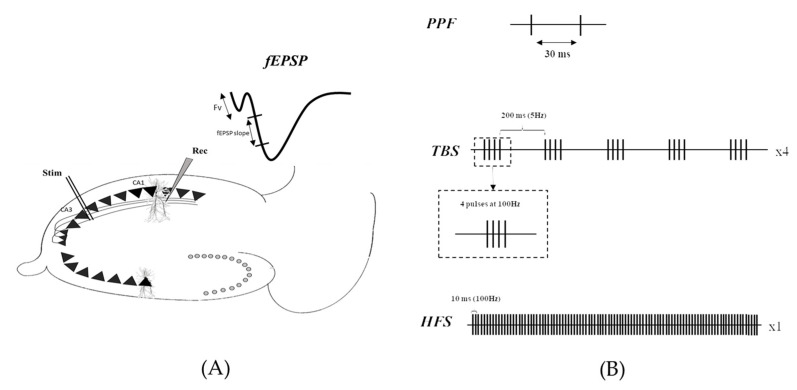
(**A**) Experimental setup, showing stimulating (Stim) and recording (Rec) electrodes placed in the stratum radiatum of the Schaeffer collateral and CA1 area, respectively. (**B**) Stimulation pattern of either HFS or TBS used to induce LTP.

As previously described [37], transverse hippocampal slices (400 µm thick) were prepared using a tissue chopper (McIlwain^®^). Briefly, field excitatory postsynaptic potentials (fEPSPs) were recorded from the CA1 area using glass micropipettes following stimulation of the Schaffer collateral axons with the bipolar tungsten electrode (Figure 4A). fEPSPs are commonly the first-in-use method to enable rapid and easy sampling of population synaptic responses resulting from glutamatergic transmission within hippocampal slice preparations [38].

Basal synaptic transmission was assessed through input/output (I/O) curves consisting of stimulation of increasing intensities (600, 800 and 1000 µA).

In addition, pre-synaptic changes were investigated using the paired-pulse facilitation (PPF) protocol, described as two electrical stimuli applied at 30 ms intervals (Figure 4B). Stimulation intensity was set to elicit a first fEPSP slope of 0.05 mV/s. This experimental procedure allows us to avoid bias in the interpretation of the results due to a difference in the basal synaptic transmission level.

The ability of the CA3-CA1 synapse to undergo long-term plasticity was assessed through LTP recordings. The stimulation intensity was set to elicit an fEPSP slope of 0.1mV/s. Following a stable 15 min baseline (0.1 Hz test pulse), LTP was induced using either HFS (100 pulses at 100 Hz) or TBS (5 bursts at 5 Hz consisting of 4 pulses at 100 Hz—and repeated 4 times at 0.1 Hz, Figure 4B) protocols.

### 4.3. Data Analyses

An index of synaptic efficacy (ISE) corresponding to the fEPSP/Fv ratio was calculated to compare basal synaptic transmission based on I/O curves. PPFs were analyzed using a facilitation index determined as the ratio of the slope of the second fEPSP to the slope of the first one (fEPSP2/fEPSP1).

The last 15 min of LTP recordings, reflecting its magnitude, were used for statistical analysis. In addition, for the TBS protocol, the trapezoidal method was used to measure the area under curve (AUC) of responses of the first five bursts. We then evaluated the potentiation of each burst relative to the first one (AUCn/AUC1) to assess the efficacy of the TBS conditioning. On-line acquisition and off-line analyses of bursts AUC and fEPSP slopes were performed using WinLTP^®^ software [39]. A fixed 1 ms cursor was placed just after the fiber volley, thus allowing the software to calculate the fEPSP slope along the linear zone [40].

### 4.4. Statistical Analyses

ANOVA for repeated measures was used to compare I/O curves with the last 15 min of LTP recordings between groups. PPF and the medians of the last 15 min of LTP recordings of each group were compared using the Mann–Whitney U test. In addition, a univariate test was used to compare the medians of the last 15 min of LTP magnitude of each group, with the value of 100% (Wilcoxon signed-rank test). Finally, the CVs were compared using a one-way ANOVA. All statistical analyses were performed using R^®^ software and graphs were drawn using GraphPad Prism software version 8 (GraphPad Software Inc., San Diego, CA, USA).

## 5. Conclusions

To conclude, Yilmazer-Hanke stated that, “studying a certain strain for neuronal correlates of learning and memory mainly tells us something about this particular strain, but not necessarily about this species or other species” [41]. In line with this way of thought, our results underline the importance of the choice of mice strains before starting experiments and, above all, invite us to pay attention to the mice’s backgrounds when interpreting the results. Our results encourage the use of C57BL/6 background mice (which exhibit a higher magnitude of TBS-LTP) to investigate the alteration of synaptic plasticity, whereas NMRI strains would be more appropriate when LTP enhancement is expected. Furthermore, the intra-strain level of individual variability (higher either in C57BL/6 or in NMRI for, respectively, the magnitude of TBS- or HFS-LTP) should also be considered when designing a research protocol.

## Figures and Tables

**Table 1:** Summary of phenotypic properties and main electrophysiological results in C57BL/6 and NMRI mice.

Zootechnical Parameters			
Strain	C57BL6/Rj	NMRI	
Genetics	Inbred	Outbred	
Coat	Black	White (albino)	
Litter size at birth	6.53	14.8	Janvier Labs 2011 data
Median life span (months)	27–31	17	Gower & Lamberty, 1993
Spatial behavioral performance (Morris water maze)			
Learning rate	C57BL6 vs. NMRI		
Escape latency	No difference		Klapdor et al., 1996; Salari et al., 2018
	C57BL6 < NMRI		Vicens et al., 1999; Vicens et al., 2002
Memory performance	C57BL6 vs. NMRI		
Time spent in target quadrant	No difference		Vicens et al., 2002; Salari et al., 2018; Klapdor et al., 1996; Vicens et al., 1999
Platform crossing	No difference		Salari et al., 2018; Klapdor et al., 1996
(Ex vivo) hippocampal synaptic plasticity			
HFS-LTP	C57BL6	NMRI	
Magnitude (%)	143 (+/−4)	149 (+/−18)	No difference
Coefficient of variation (%)	12%	41%	C57BL/6 < NMRI
TBS-LTP	C57BL6	NMRI	
Magnitude (%)	169 (+/−10)	127 (+/−4)	C57BL/6 > NMRI
Coefficient of variation (%)	26%	14%	C57BL/6 > NMRI

## Data Availability

The original contributions presented in the study are publicly available. The data can be found here: [https://doi.org/10.6084/m9.figshare.19596610] accessed on 14 April 2022.
